# Metastructure-enabled scalable multiple mode-order converters: conceptual design and demonstration in direct-access add/drop multiplexing systems

**DOI:** 10.1515/nanoph-2025-0364

**Published:** 2025-12-01

**Authors:** Zhenzhao Guo, Weike Zhao, Shengbao Wu, Yunfeng Lai, Shuying Cheng, Daoxin Dai

**Affiliations:** Institute of Micro/Nano Devices and Solar Cells, School of Physics and Information Engineering, 12423 Fuzhou University, Fuzhou 350108, China; State Key Laboratory for Extreme Photonics and Instrumentation, College of Optical Science and Engineering, Zhejiang University, Hangzhou 310058, China; Photonics Information Innovation Center, Hebei Provincial Center for Optical Sensing Innovations, College of Physics Science and Technology, Hebei University, Baoding 071002, China

**Keywords:** multimode silicon photonics, metastructures, mode conversion, subwavelength metamaterial, mode division multiplexing

## Abstract

The rapid advancement of multimode photonic technologies, optical computing, and quantum circuits, leveraging higher-order modes, necessitates the development of on-chip multiple mode-order converters (MMOCs). However, existing schemes face limitations in traffic capacity, polarization-dependence, and scalability. Herein, we propose a novel highly scalable MMOC design framework enabled by subwavelength grating (SWG) metastructures. By integrating SWG arrays into a taper-tailored multimode waveguide, the design synergizes coherent scattering and beam shaping to achieve efficient target-supermode excitations and precise phase controls, simultaneously. In this way, the target MMOC can be realized according to the functional requirements of mode manipulations by optimizing the metastructures. Experimentally fabricated devices exhibit ILs < 1.85 dB and CTs < −12.5 dB across (22 or 50) nm bandwidths, with a polarization-independent quad-mode operation. Notably, the dual-pair mode exchanging MMOC pioneers simultaneous TE_0_↔TE_2_ and TE_1_↔TE_3_, doubling exchange efficiency over conventional single-pair solutions. Integrated into a direct-access mode add/drop system (DAMAD), TE_0_/TE_1_ dual-mode add/drop operations achieve ILs < 4.5 dB and CTs < −15.5 dB across 41 nm bandwidth. Thereupon, clear eye diagrams at 32/64 Gbps operations demonstrate the capability for the high-speed optical communication. The proposed concept offers a novel strategy for on-chip multiple mode manipulations, with transformative potential in higher-order modes based optical communications.

## 1 Introduction

Recent advancements in on-chip integrated photonics have witnessed growing interest in exploiting higher-order modes within the multimode waveguide as critical optical information carriers. These spatially orthogonal eigenmodes, characterized by unique electromagnetic field distributions, offer unprecedented opportunities for enhancing data transmission capacity through mode-division multiplexing (MDM) schemes [1], [2], [3]. Beyond telecommunications, such mode engineering has catalyzed innovations across quantum photonic circuits [4], [5], neuro-inspired computing [6], biochemical sensing devices [7], optical switches [8], interpath routing [9], and nonlinear optical phenomena manipulation [10]. For example, TE_1_ mode coupled with the fundamental mode TE_0_ via shared decay channels in the bus waveguide forms Friedrich–Wintgen bound states in the continuum to suppress parasitic loss and enhance quality factor [11]. For quantum optics, L-T. Feng et al. demonstrated the first implementation of a transverse mode-encoded 2-qubit controlled-NOT (CNOT) gate on a silicon photonic chip using the higher-order transverse mode (TE_1_ mode) [12]. This CNOT gate entangles two separable transverse mode qubits (TE_0_ and TE_1_), generating Bell states with an average state fidelity of 0.89 ± 0.02. Y. Zheng et al. presented the first experimental demonstration of a multichip, multidimensional quantum entanglement network based on silicon photonic integrated chips [13]. Such a network leverages hybrid multiplexing (wavelengths, paths, polarizations, and modes) to distribute multidimensional entangled states across multiple chips connected with a few-mode fiber (FMF). For the mode-encoding, the mode (De)multiplexers (DeMUXs) and multimode chip-fiber edge couplers transform on-chip path-encoded states (e.g., |0⟩, |1⟩, |2⟩, |3⟩) into hybrid polarization-mode–encoded states in FMFs, significantly increasing channel capacity.

Central to above-mentioned applications lies the critical functionality of higher-order mode manipulation. Mode-order converters serving as classical and essential components for basic conversions between the given mode and desired mode [14] have been extensively investigated over the past decade [15], [16], [17]. However, single-channel operation of conventional mode-order converters increasingly limits their utility in advanced photonic systems, which requires parallel and multidimensional mode processing [18]. Building upon this foundation, multiple mode-order converters (MMOCs) that realize multichannel mode manipulations in parallel are desired, which can be used for passive multichannel signal switching and routing [19]. Currently, MMOCs predominantly leverage one of phase matching, beam shaping, and coherent scattering as the multiple mode converting mechanisms [20]. For instance, directional couplers (DCs) are typical configurations to realize on-chip MMOCs. Nevertheless, owing to the strict phase-matching condition between two coupling waveguides, DCs-based MMOCs usually suffer from the narrow bandwidth (∼10 nm) or sophisticated fabrication processes (multistep etching and metal depositions) [21], [22]. By contrast, coherent scattering can broaden the operational bandwidth for MMOCs, which essentially excites supermodes by interference principle. A scalable MMOC model is proposed by using subwavelength metamaterial structures to manipulate the multimode excitations/interferences, which can realize mode exchange between the input *i*-th and *j*-th order modes (mode exchangers) [23]. This scalable approach experimentally demonstrates four types of MMOCs, including TE_0_-TE_1_/TE_1_-TE_0_, TE_0_-TE_2_/TE_2_-TE_0_, TE_0_-TE_3_/TE_3_-TE_0_, and TE_1_-TE_2_/TE_2_-TE_1_ with all devices featuring an ultracompact footprint of < 5.2 μm, and the best operational bandwidth for keeping loss (IL) < 0.3 dB and crosstalk (CT) < −12 dB is 89 nm. As an alternative approach, beam shaping, which essentially splits input mode into modal components with different phases and further combines these components into the desired mode, has been investigated. In Ref. [24], two MMOCs are optimized by the adjoint inverse design method, where the formed Mach–Zehnder interferometer-like structure can achieve TE_0_-TE_1_/TE_1_-TE_0_/TE_2_-TE_2_ mode conversions. The working bandwidth is as large as 100 nm for IL < 1.7 dB and crosstalk (CT) < −16.5 dB. Despite remarkable progress in MMOCs, these approaches exhibit polarization-dependent operations (mostly for TE modes), which is a fundamental limitation arising from the high-index-contrast of silicon-on-insulator (SOI) platform. As to the channel capacity, current exchangers are only capable of facilitating the mode exchange between a single pair of modes. Furthermore, most reports have limited scalability and generally necessitate configuration redesigns if the mode-order conversion function is altered, particularly when input and output modes are changed. Therefore, achieving high performance MMOCs with multichannel capabilities, polarization-independent operation and high scalability still poses a significant challenge.

Recently, subwavelength grating (SWG) metamaterials have emerged as a novel platform for optical design, utilizing periodic dielectric nanostructures with spacing far below optical wavelengths to enable unprecedented control over light behavior. These metamaterials overcome diffraction limits by functioning as an effective homogeneous medium, enabling localized refractive index manipulation [25]. The design flexibility of SWG meta-structures provides researchers with broad design possibilities, allowing on-chip devices to achieve multifunctionality while maintaining ultracompact integration. Their unique properties have driven widespread adoption in silicon multimode photonics, including applications in MOCs and MMOCs [26], [27]. Inverse-designed “QR-code” MMOCs exploiting the phase shaping, typically implemented as meta-structures [28], [29], demonstrate exceptional scalability for realizing multiple mode conversions with arbitrary target mode orders. Beyond that, inverse-designed metastructures exhibit high design scalability, which can be extended to the development of a wide variety of silicon multimode devices. Examples include multimode power splitters, waveguide crossings, and mode/wavelength demultiplexers [30], [31], [32], [33]. This flexibility highlights their potential for enabling advanced integrated photonic circuits with enhanced functionality and compact footprints. Nevertheless, performance remains critically dependent on initial geometry configurations. Meanwhile, these MMOCs exhibit inherent polarization dependence, primarily operating in TE modes.

In this paper, we propose and demonstrate a novel design framework for scalable on-chip MMOCs by leveraging the SWGs metastructures. The designed physical model is engineered by SWGs arrays to form synergistic coherent scattering and beam shaping regions within a taper-tailored multimode waveguide, significantly enhancing the degree of freedom in mode-profile manipulations. Unlike previously reported MMOCs, which primarily rely on a single mode conversion mechanism, this approach allows for simultaneous efficient target-supermode excitation and precise phase control of modal components within a single device. By optimizing the meta-structure, multi-input modes can be converted into multi-output modes according to the functional requirements of the MMOC. As a proof of concept, four types of MMOCs are designed and demonstrated with compact device lengths ranging from 9.234 μm to 12 μm. For the measurements, these MMOCs show ILs < 1.85 dB and CTs < −12.5 dB across 22–50 nm bandwidths. In terms of functionality, a polarization-independent multiple mode conversion of TE_0_-TE_2_/TE_1_-TE_3_/TM_0_-TM_2_/TM_1_-TM_3_ is realized. More importantly, we first demonstrate a dual-pair mode-exchanger that can facilitate a two-mode exchange process (TE_0_↔TE_2_ and TE_1_↔TE_3_), and this feature doubles the mode swap efficiency compared with previously reported MMOCs. Such a device can significantly improve the add-drop capacity of direct-access multiplexing network systems.

## 2 Theoretical model, principle, and optimization

### 2.1 Configuration and operating principle

**Figure 1:**
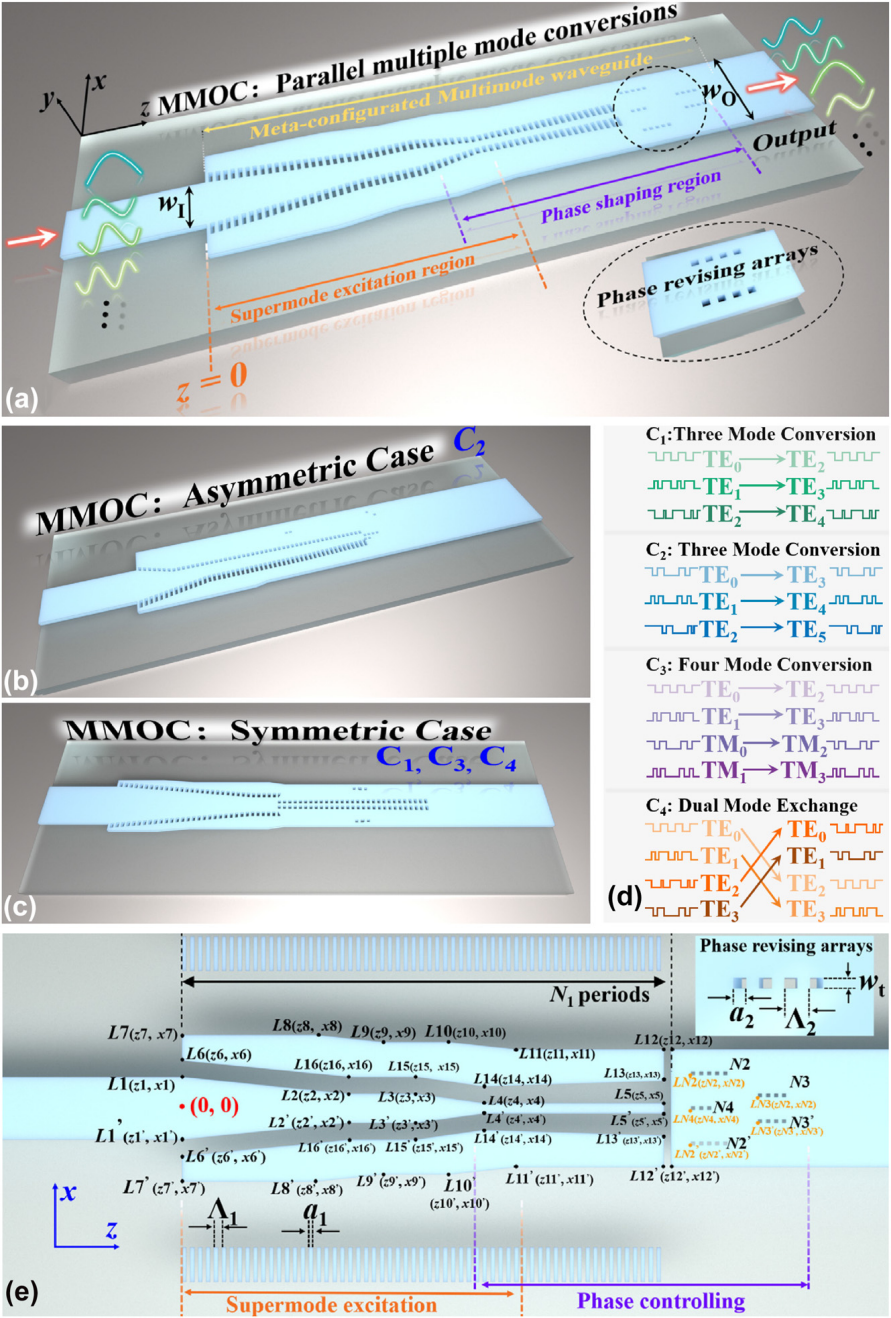
3D physical model of the proposed meta-configurated MMOC concept: (a) basic concept; (b) converter C2 in the asymmetric case; (c) converters C1, C3, and C4 in the symmetric case, together with enlarged views of etched grating arrays. The model is covered by upper silica claddings, which is intentionally excluded from the figures to improve graphical readability. (d) The functions of designed MMOCs. (e) Schematics of the model, where the geometry is denoted by coordinates.

Figure 1 shows the physical model of the proposed MMOC concept, including four proof-of-concept devices, which are named as **C**_**1**_, **C**_**2**_, **C**_**3**_, and **C**_**4**_, respectively, according to the MMOC functionality. This model is designed based on a standard 220-nm SOI platform with *n*_Si_ = 3.476, a 2-μm buffer oxide SiO_2_ layer with *n*_SiO2_ = 1.444, and covered by a 2.2-μm SiO_2_ cladding layer. The *N*-channel mode conversion function of each MMOC is labeled in Figure 1(d). In principle, the symmetry of the converter is determined by the parities of input and output modes. Different parities of input and output modes necessitate symmetry breaking along the *x*-direction in the model, resulting in an asymmetric configuration. By contrast, a symmetric structure can realize mode conversion between the same parities, which reduces ∼50 % structural parameters, significantly lowering the optimization complexity. In light of this, converter C_1_, C_3_, and C_4_ are in symmetric case where input and output modes are both even (TE_0_, TE_2_, TE_4_, TM_0_, TM_2_) or odd (TE_1_, TE_3_, TE_5_, TM_1_, TM_3_) modes, and C_2_ is in asymmetric case due to the opposite mode parity. The presented MMOC concept can be modeled into an input waveguide, an output waveguide etched by SWGs phase revising arrays, a tapered multimode waveguide formed by three tapers, and *N*_1_-period embedded SWG meta-arrays, as shown in Figure 1(e). Structural parameters of the MMOC physical model are denoted by coordinates. For example, the widths of input and output waveguide *w*_I_ and *w*_O_ can be obtained by *x*1–*x*1^’^ and *x*12–*x*12^’^, respectively. The position of etched meta-arrays is indicated by *LN*2, *LN*2^’^, *LN*3, *LN*3^’^, *LN*4, with period numbers labeled nearby. For all these etched arrays, they have the same pitch length of Λ_2_ and the same duty cycle of *a*_2_/Λ_2_ in this work. Moreover, one has *z*2 = *z*16, *z*2^’^ = *z*16^’^, *z*4 = *z*14, *z*4^’^ = *z*14^’^, and *z*5 = *z*13 = *z*5^’^ = *z*13^’^ = *z*12 = *z*12^’^ in this model.

**Figure 2:**
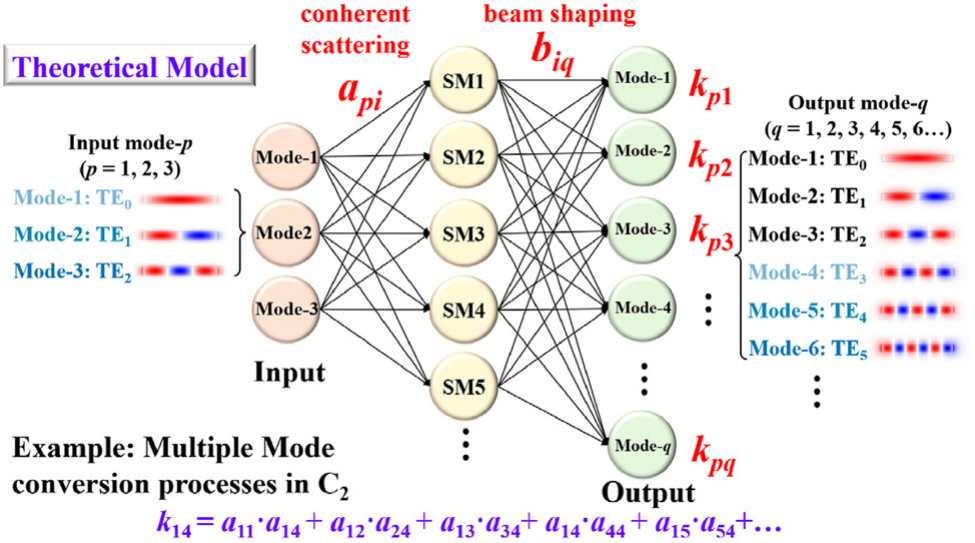
Theoretical model of the proposed meta-configurated MMOC concept: the mode conversion matrix.

By using the SWG meta-arrays to engineer the multimode waveguide, synergistic supermode excitation (SE) and phase controlling (PC) metastructures are formed, which can manipulate the optical field to conduct the multiple mode conversions. For each input mode, different supermodes are gradually excited through the SE region by the principle of coherent scattering, in which the target mode field distribution is the superposition of these supermodes. Meanwhile, the SE triggers concurrent beam shaping in the PC region, where the phase differences between modal components (or say mode beams) of the target mode are revised to attain a π phase difference. In this way, all input modes can be converted to the corresponding desired modes in the MMOC model by optimizing the metastructures. For theoretical analysis, the principle of MMOC can be modeled by the mode conversion matrix (MCM). Here, we take converter C_2_ as an example for easy understanding. As shown in Figure 2, SE region excites supermodes (abbreviated SM1, SM2, … in the following) with different amplitudes, for input mode-*p* (*p* = 1, 2, 3). In this process, *a*_pi_ (*i* = 1, 2, 3, …) stands for the forward mode conversion coefficient between mode-*p* and supermodes. Next, the excited supermodes are converted into different output eigenmodes mode-*q* (*q* = 1, 2, 3, …) with different energy fractions, by the phase revising process in PC region. Likewise, *b*_iq_ represents the forward mode conversion coefficient between supermodes and output mode-*q*. As such, MCM along the transmission direction can be expressed as below.

(1) $$\begin{array}{ll} & \left[k_{p1}\;k_{p2}\;k_{p3}\;\ldots \;k_{pq}\right]=\left[a_{p1}\;a_{p2}\;a_{p3}\;\ldots \;a_{pi}\right]\\ & \;\;\times \left[\begin{array}{ccccc} b_{11} & b_{12} & b_{13} & \ldots & b_{1q}\\ b_{21} & b_{22} & b_{23} & \ldots & b_{2q}\\ b_{31} & b_{32} & b_{33} & \ldots & b_{3q}\\ \vdots & \vdots & \vdots & \ddots & \vdots \\ b_{i1} & b_{i2} & b_{i3} & \ldots & b_{iq} \end{array}\right] \end{array}$$

where *k*_*pq*_ is the conversion coefficient between the given mode-*p* and the desired mode-*q*. The modulus square of coefficients signifies the efficiency of mode conversion. In order to achieve C_2_, the following relationship should be satisfied

(2) $$\left[\begin{array}{c} K_{\text{Out}4}\\ K_{\text{Out}5}\\ K_{\text{Out}6} \end{array}\right]=\left[\begin{array}{ccc} k_{14} & 0 & 0\\ 0 & k_{25} & 0\\ 0 & 0 & k_{36} \end{array}\right]\left[\begin{array}{c} K_{\text{In}1}\\ K_{\text{In}2}\\ K_{\text{In}3} \end{array}\right]$$

in which *K*_Out3_, *K*_Out4_, and *K*_Out5_ are the normalized amplitudes of desired output mode-4, -5, and -6, i.e., TE_3_, TE_4_, and TE_5_ modes, respectively. Moreover, *K*_In1_, *K*_In2_, and *K*_In3_ are the amplitudes of input mode-1, -2, and -3, i.e., TE_0_, TE_1_, and TE_2_ modes, respectively. As such, |*k*_14_|^2^, |*k*_25_|^2^, and |*k*_36_|^2^ require concurrent maximization under the metastructures optimizations.

From the perspective of beam shaping, a higher-order mode can be thought of as a superposition of multiple adjacent beams. When an etched SWGs array is introduced around one beam, the effective refractive index distribution is locally modified according to the effective medium theory [34]. Consequently, a small phase shift can be accomplished for the beam. By accumulating and revising these shifts, a target phase difference of π between adjacent beams can be achieved to form the desired higher-order mode. Here, we use five SWGs arrays to perform the phase revising in our design, and the reason is explained as follows. For MMOCs C_1_, C_3_, and C_4_, the highest-order desired mode is TE_4_ mode, which comprises five beams in total. A five-SWGs array can thus cover all beams and simultaneously adjust the phase differences for three or four mode conversions. For MMOC C_2_ under a three-mode conversion, the highest-order desired mode is TE_5_ mode with six beams. The five SWGs arrays can guarantee the phase shifting for five beams, which in turn effectively revises the phase difference for all six beams as they are adjacent. In such a case, the phase difference for all six beams still can be tuned.

### 2.2 Optimizations and results

Based on the MCM, the C_2_ is designed as follows. The SWG meta-arrays can be modeled as an equivalent medium by Rytov’s formula in the mode-field calculations [35]:

(3) $$n_{\text{meta }-\text{ arrays}}^{2}\approx \frac{a}{\Lambda }\cdot n_{\text{Si}}^{2}+\left(1-\frac{a}{\Lambda }\right)\cdot n_{\text{Si}{\text{O}}_{2}}^{2}$$

For the eigen mode-field, the finite-element method (FEM) is used to obtain electric E and magnetic H fields [36]. Afterward, the eigenmode expansion theory [37] is performed to calculate the mode conversion coefficients for MCM, to conduct the initial parameters choice. For launched mode-1, -2, and -3, i.e., TE_0_, TE_1_, and TE_2_ modes, mode conversion coefficients are calculated and shown in Table 1.

**Table 1:** Calculated mode conversion coefficients for C_2_.

TE_0_ input	*a*_11_	*a*_12_	*a*_13_	*a*_14_
	0.100241 − 0.15322i	−0.146937 − 0.054985i	0.165976 + 0.082762i	−0.842396 + 0.251638i
	*a*_15_	*a*_16_	*a*_17_	*a*_18_
	−0.249046 − 0.187935i	−0.10336 + 0.061935i	−0.064478 + 0.086431i	−0.074948 − 0.033824i
	*b*_14_	*b*_24_	*b*_34_	*b*_44_
	0.103949 − 0.096142i	0.146432 + 0.173414i	0.06304 − 0.351746i	−0.710166 + 0.438038i
	*b*_54_	*b*_64_	*b*_74_	*b*_84_
	0.003042 + 0.226137i	−0.089969 + 0.039748i	−0.032357 + 0.029608i	−0.001799 + 0.07654i
TE_1_ input	*a*_21_	*a*_22_	*a*_23_	*a*_24_
	−0.0844952 − 0.162835i	−0.11934 − 0.296492i	−0.005884 − 0.44724i	0.225601 − 0.021559i
	*a*_25_	*a*_26_	*a*_27_	*a*_28_
	0.031739 − 0.421532i	−0.399283 + 0.213998i	−0.224142 + 0.163034i	−0.287032 − 0.059239i
	*b*_15_	*b*_25_	*b*_35_	*b*_45_
	−0.110953 − 0.132907i	−0.0519813 − 0.273267i	−0.0661801 − 0.306796i	−0.249691 − 0.0443426i
	*b*_55_	*b*_65_	*b*_75_	*b*_85_
	0.0252396 − 0.472877i	0.415804 + 0.136813i	0.325437 − 0.0490269i	0.241595 − 0.193477i
TE_2_ input	*a*_31_	*a*_32_	*a*_33_	*a*_34_
	−0.092197 − 0.088538i	0.337654 + 0.155419i	−0.334449 − 0.145009i	−0.184418 − 0.0613076i
	*a*_35_	*a*_36_	*a*_37_	*a*_38_
	−0.0969664 + 0.462156i	−0.149011 + 0.0279027i	−0.402875 − 0.291955i	−0.327016 − 0.115452i
	*b*_16_	*b*_26_	*b*_36_	*b*_46_
	0.0395943 − 0.0450513i	−0.268447 + 0.0051849i	0.133182 + 0.118719i	0.122417 + 0.0933039i
	*b*_56_	*b*_66_	*b*_76_	*b*_86_
	−0.338669 + 0.478922i	0.125387 + 0.206449i	0.544466 + 0.125428i	0.271946 + 0.12417i

**Figure 3:**
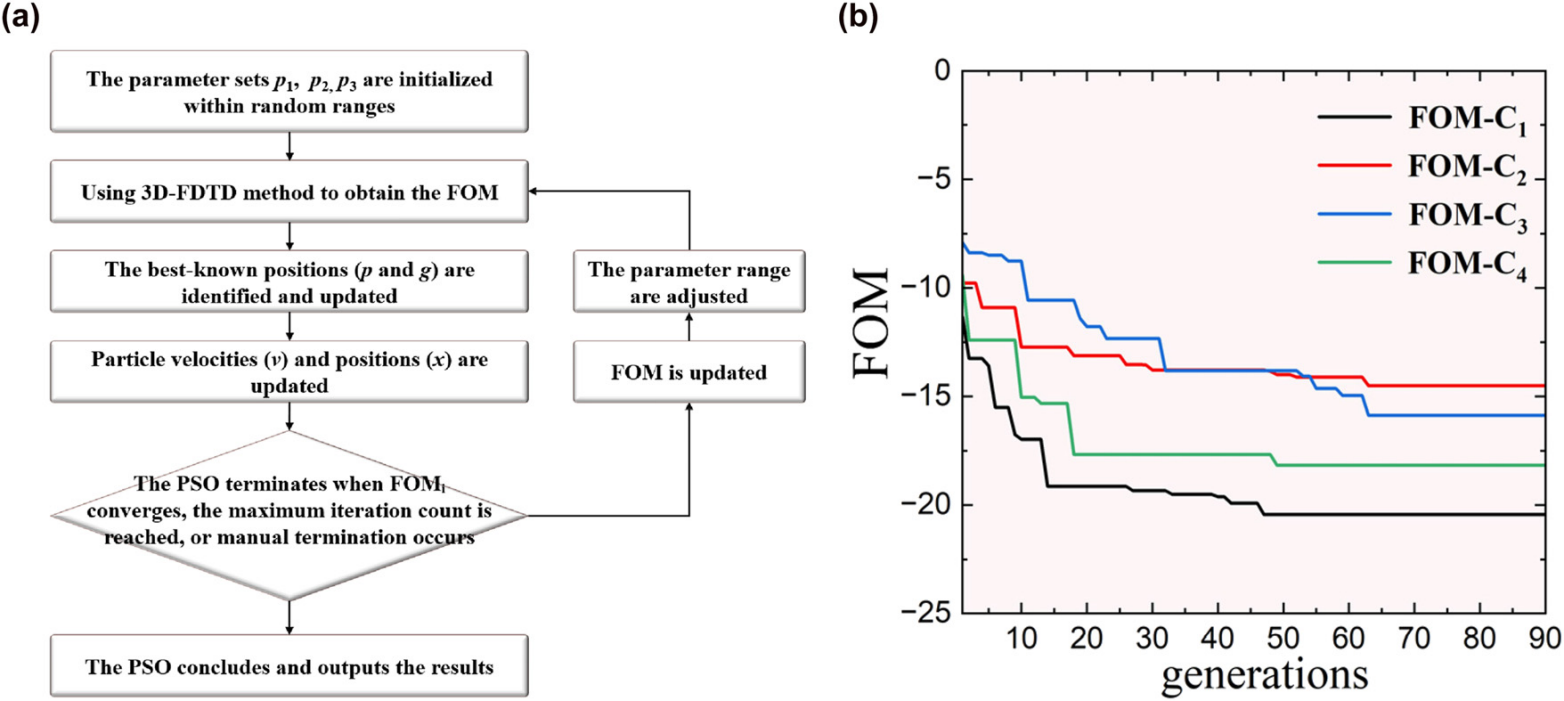
The PSO optimization process: (a) Flowchart of the PSO optimization; (b) calculated FOM with varied number of generations in the PSO optimization.

By substituting coefficients from Table 1 into Equation (1), *k*_14_ = 0.5621 − 0.7369i, *k*_25_ = −0.8208 + 0.2181i, and *k*_36_ = −0.6135 − 0.6348i can be achieved, with conversion efficiencies of *|k*_14_|^2^ = 85.9 %, |*k*_25_|^2^ = 72.12 %, and |*k*_36_|^2^ = 77.94 %. In this scenario, corresponding *w*_I_ and *w*_O_ are 1.62 μm and 3 μm, respectively, and the other key cross section parameters set ***p***_**1**_ are summarized as following: *x*2 = *x*3 = 0.28 μm, *x*15 = *x*16 = 0.44 μm, *x*2^’^ = *x*3^’^ = −0.28 μm, *x*7 = *x*8 = 1.8 μm, *x*9 = *x*10 = 1.6 μm, *x*7^’^ = *x*8^’^ = −1.35 μm, *x*9^’^ = *x*10^’^ = −1.25 μm, *x*15^’^ = *x*16^’^ = −0.63 μm, *x*4 = *x*5 = 0.23 μm, *x*13 = *x*14 = 0.38 μm, *x*4^’^ = *x*5^’^ = −0.15 μm, and *x*13^’^ = *x*14^’^ = −0.52 μm, *x*11 = *x*12 = 1.8 μm, and *x*11^’^ = *x*12^’^ = −1.2 μm. As to the SWG meta-arrays, period length Λ_1_ and filling-factor *a*/Λ_1_ are fixed as 200 nm and 50 %. Based on this prototype design, the particle swarm optimization (PSO) method [38] is adopted to carry out the final optimization. The size of each generation is set to be 30, and the figure of merit (FOM) for C_2_ is formulated as

(4) $$\begin{array}{ll} FOM & =\max\left\{{CT}_{14}^{\text{TE}},{CT}_{25}^{\text{TE}},{CT}_{36}^{\text{TE}}\right\}\\ & \;+\alpha \cdot \max\left\{{IL}_{14}^{\text{TE}},{IL}_{25}^{\text{TE}},{IL}_{36}^{\text{TE}}\right\} \end{array}$$

where *α* = 2 is the weight coefficient to make a reasonable trade-off between ILs and CTs. In FOM, the IL and CT are defined as

(5) $$IL\left(dB\right)=-10\cdot \log\frac{P_{q}^{\text{O}}}{P_{q}^{\text{I}}}$$

(6) $$CT\left(dB\right)=\max\left\{10\cdot \log\frac{P_{ohter}^{\text{O}}}{P_{q}^{\text{O}}}\right\}$$

where $P_{y}^{x}$ represents the optical power collected at *x*-port (*x* = I: input port and *x* = O: output port) with *y*-mode (*y* = *p*: input mode-*p*, *y* = *q*: target mode-*q*, and *y* = other: other undesired modes), for both TE and TM polarized light. For the simulations, the three-dimensional finite-difference time-domain (3D-FDTD) [39] is performed to calculate ILs and CTs. Specially, symmetric (asymmetric) boundary condition along *y*-direction is used for input TE (TM) modes to save ∼50 % simulation time, according to the dominant electric field component of input modes. In the PSO algorithm, the position and velocity of the particle can be adjusted using the equations provided below [38]:

(7) $$\begin{array}{ll} v_{t+1} & =\omega \cdot v_{t}+c_{1}\cdot rand\left(\right)\cdot \left(p_{t}-x_{t}\right)\\ & \;+c_{2}\cdot rand\left(\right)\cdot \left(g_{t}-x_{t}\right) \end{array}$$

where *c*_1_ and *c*_2_ are the cognitive factor and social factor, respectively, also known as the acceleration constants. Moreover, *ω* is the inertia weight, which controls the exploration scope, and rand() denotes a uniformly distributed random number within the interval [0,1]. Correspondingly, the PSO flow is described in Figure 3(a).

In the optimization, *z*1, *z*1^’^, *z*6, *z*6^’^, *z*7, *z*7^’^ are fixed as 0, and the initial guessing range for parameter set ***p***_**2**_ of *z*2/*z*2^’^, *z*3/*z*3^’^, *z*4/*z*4^’^, *z*5/*z*5^’^, *z*8/*z*8^’^, *z*9/*z*9^’^, *z*10/*z*10^’^, and *z*11/*z*11^’^ is set to be [1.5, 4.5], [3, 6.5], [4.5, 8], [6.5, 10], [0.5, 2.5], [2.5, 5], [5, 7.5], and [7.5, 10] μm. As to the *y*-axis parameter set ***p***_**1**_ mentioned above, the initial guessing range is ***p***_**1**_ ± 0.2 μm. For the fully etched phase revising array, the initial guessing for parameter set ***p***_**3**_ of *zN*2/*zN*2^’^/*zN*3/*zN*3^’^/*zN*4, *xN*2, *xN*2,^’^ *xN*3, *xN*3,^’^ and *xN*4 is chosen to be [10, 12.5], [1.3, 1.8], [0.8, 1.3], [0.3, 0.8], [−0.2, 0.3], and [−0.7, −0.2] μm. The parameter range is updated accordingly as the FOM remains unchanged over 20 generations. Finally, the optimization for MMOC ceases when the FOM with a parameter update no longer shows improvement. The last runs of the PSO optimizations for all devices are shown in Figure 3(b). The FOMs become saturated after 42–62 generations for all devices. For converters C_1_, C_2_, C_3_, and C_4_, optimal parameters are shown in Table S1 (Supplementary Material).

**Figure 4:**
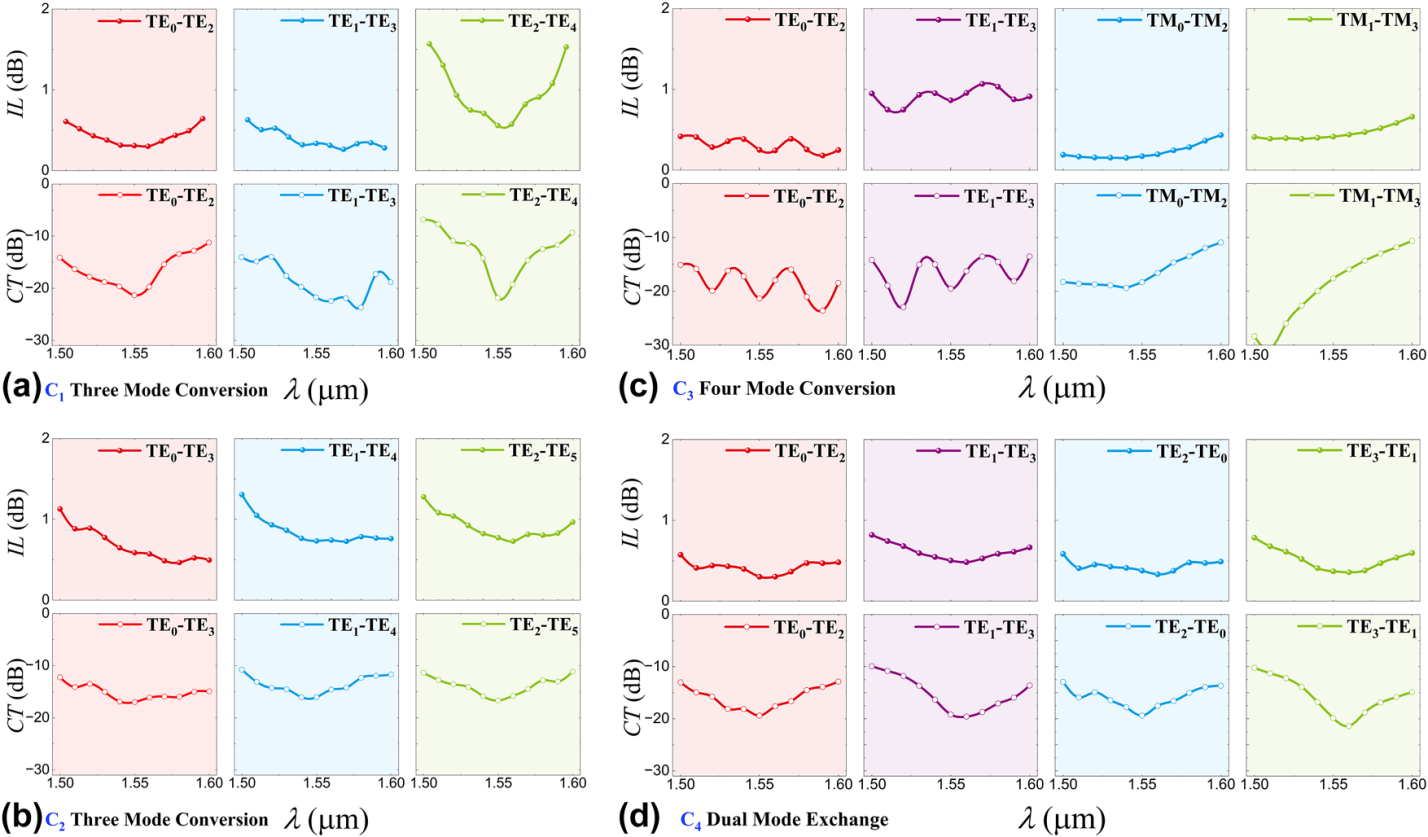
Calculated ILs and CTs spectra for optimal MMOC (a) C_1_, (b) C_2_, (c) C_3_, and (d) C_4_.

The spectral performance of converters C_1_–C_4_ is calculated through 3D-FDTD simulations using optimized design parameters, as illustrated in Figure 4. MMOC C_1_ exhibits triple-mode conversion capabilities at 1,550 nm, with TE_0_-TE_2_, TE_1_-TE_3_, and TE_2_-TE_4_ mode conversions showing ILs of 0.3 dB, 0.33 dB, and 0.55 dB, respectively, paired with CT levels of −21.53 dB, −21.75 dB, and −21.87 dB. Notably, the IL and CT @1550 nm of TE_1_-TE_3_ conversion are even lower than those of some scalable single mode-order converter using SWGs (IL = 0.46 dB/CT = ∼ −17 dB and IL = 0.53 dB) [40], [41], metasurface (IL = ∼1.316 dB/CT = ∼ −10.1 dB) [42], and tapered metal cap (IL = 2 dB/CT = −16 dB) [43]. Operating bandwidth across a 60 nm range (1,520–1,580 nm), where all three conversions maintain ILs under 1 dB and CTs below −10.5 dB. At 1,550 nm central wavelength, MMOC C_2_ achieves ILs of 0.58 dB, 0.73 dB, and 0.77 dB for TE_0_-TE_3_, TE_1_-TE_4_, and TE_2_-TE_5_ mode conversions, respectively, accompanied by CT values of −16.99 dB, −16.05 dB, and −16.68 dB. As the ILs/CTs < 1 dB/−10.5 dB, the operational bandwidth spans over 80 nm (from 1,520 nm to 1,600 nm) for a triple mode converting process. For MMOC C_3_, both polarization states maintain ILs below 1 dB and CT suppression stronger than −10.5 dB across a 100 nm spectral window (1,500–1,600 nm), demonstrating broader operational bandwidth compared to previously reported polarization-independent TE_0_-TE_1_ mode converters employing inverse design strategies [44]. Simulated ILs/CTs at 1,550 nm exhibit 0.25 dB (TE_0_-TE_2_), 0.86 dB (TE_1_-TE_3_), 0.17 dB (TM_0_-TM_2_), and 0.41 dB (TM_1_-TM_3_) conversion efficiencies, with corresponding CT levels reaching −21.35 dB, −19.5 dB, −18.31 dB, and −17.6 dB, respectively. Regarding MMOC C_4_, also a mode exchanger realizing a dual-pair mode-exchange process, under the same operational thresholds (IL < 1 dB and CT < −10.5 dB), the converter maintains 90 nm bandwidth (1,510–1,600 nm) across both TE_0_↔TE_2_ and TE_1_↔TE_3_ mode exchanges, which is even larger than those of single-pair mode exchangers [21], [22], [29]. At the central wavelength of 1,550 nm, the ILs/CTs for TE_0_-TE_2_, TE_2_-TE_0_, TE_1_-TE_3_, and TE_3_-TE_1_ mode conversions are 0.3 dB/−19.4 dB, 0.37 dB/−19.41 dB, 0.5 dB/−19.16 dB, and 0.36 dB/−19.88 dB, respectively.

**Figure 5:**
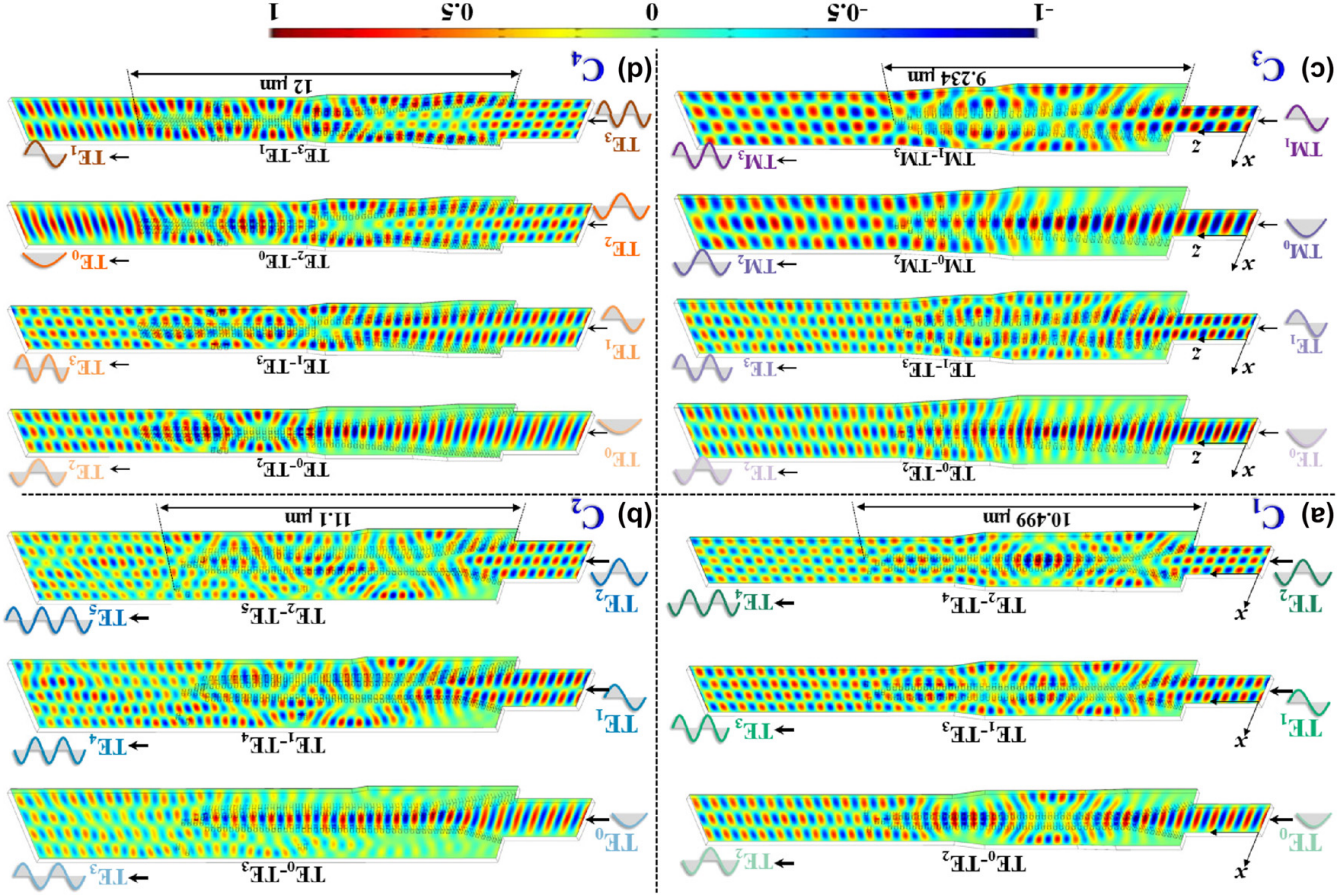
Simulated light propagations for MMOC (a) C_1_, (b) C_2_, (c) C_3_, and (d) C_4_, respectively, at the operating wavelength of 1.55 μm.

The mode conversion processes in devices C_1_–C_4_ are simulated within the SWG engineered metastructures by the 3D-FDTD method, where input modes are first transformed into supermodes through coherent scattering interference in the supermode excitation region, followed by precise beam shaping to manipulate phase differences among modal components in phase shaping region, ultimately generating desired output modes, as shown in Figure 5. From Figure 5(a), in converter C_1_, input TE_0_, TE_1_, and TE_2_ modes undergo mode excitations to form hybrid supermodes that are subsequently shaped by SWG arrays to adjust the relative phases of three modal components, achieving simultaneous TE_0_-TE_2_, TE_1_-TE_3_, and TE_2_-TE_4_ conversions. For converter C_2_ shown in Figure 5(b), the supermodes containing 4/5/6 modal components are excited through asymmetric scattering for TE_0_/TE_1_/TE_2_ inputs. These components are selectively combined via phase shaping region, realizing TE_0_-TE_3_, TE_1_-TE_4_, and TE_2_-TE_5_ conversions. It seems that the device performance of TE_2_-TE_5_ in MMOC C_2_ is poorer than those of TE_0_-TE_3_ and TE_1_-TE_4_ shown in Figure 5(b), when compared with balanced results in Figure 4(b). A detailed top view of light propagations is shown in Fig. S2 (Supplementary Material). From this top view, the TE_0_-TE_3_ and TE_1_-TE_4_ mode conversions also show some poor mode field distributions at certain positions, which are marked by red dashed lines. As to the TE_2_-TE_5_ mode conversion, some reasonable mode field distributions can be observed, which is marked by blue dashed lines. Notably, converter C_3_ illustrates a polarization-independent multiple manipulation, where TE_0_/TE_1_ and TM_0_/TM_1_ inputs, respectively, excite same supermode patterns in orthogonal polarizations, with 3 and 4 modal components being shaped into TE_2_/TM_2_ and TE_3_/TM_3_ outputs, as shown in Figure 5(c). Specifically, converter C_4_ features the dual-pair mode exchange functionality, where TE_0_↔TE_2_ and TE_1_↔TE_3_ mutual conversions are achieved. From Figure 5(d), input TE_0_, TE_1_, TE_2_, and TE_3_ modes excite supermodes whose mode field distributions are like TE_2_, TE_3_, TE_0_, and TE_1_ modes, respectively, by the SWG tailored supermode excitation metastructure. As these supermodes are being excited, they undergo phase shaping to gradually generate output TE_2_, TE_3_, TE_0_, and TE_1_ modes, enabling simultaneous two-channel mode swapping within a compact footprint of 12 μm. To confirm the scalability of the proposed design framework, an MMOC achieving TE_0_-TE_6_, TE_1_-TE_7_, and TE_2_-TE_8_ mode conversions is further designed and optimized, denoted as C_5_. The corresponding light propagations are simulated and shown in Fig. S3 (Supplementary Material). Accordingly, the calculated ILs (CTs) for TE_0_-TE_6_, TE_1_-TE_7_, and TE_2_-TE_8_ mode conversions are 1.06 dB (−9.84 dB), 1.03 dB (−10.79 dB), and 1.01 dB (−11.74 dB), respectively, at the wavelength of 1.55 μm.

For fabrication tolerance study, it is important to evaluate the impact of etching and deposition variations. In our work, the thickness of the deposited upper cladding layer is 2.2 μm, which can cover the 220-nm-thick devices totally. Thus, we only investigate the dimension variations on the under-etch case for 220-nm device layer etching (Δ*h*). Besides, we investigate the dimension variations on Δ*a* (different lengths of *a* resulting in different duty cycles of SWGs), as shown in Fig. S1 (Supplementary Material). From Fig. S1, to ensure ILs (CTs) lower than 1.5 dB (−10 dB), Δ*h* should be controlled within the range of [0, 5] nm, [0, 20] nm, [0, 25] nm, and [0, 10] nm, respectively, for MMOCs C_1_, C_2_, C_3_, and C_4_. These ranges are fairly enough for fabrication purposes since a precise etch depth can be targeted by using the etch chemistry that is unable to etch away SiO_2_, where the etch recipe stops at the buried oxide layer. Meanwhile, the same performance can still be obtained with Δ*a* varying in the range of [−10, 5] nm, [−5, 5] nm, [−10, 15] nm, and [−10, 5] nm, respectively, for MMOCs C_1_, C_2_, C_3_, and C_4_. A focused ion beam milling with beam size of <5 nm in diameter [45] can relieve the fabrication tolerance of spaced gap in SWGs. Indeed, for mass productions, a more scalable fabrication process such as 180 nm ultraviolet (UV) lithography is more favorable. However, such a fabrication technology cannot ensure the deep SWG operating condition for 1.55 µm light. Fortunately, the electron-beam lithography (EBL) is capable of defining feature sizes of 100 nm or smaller for large productions. The EBL is currently supported by the majority of wafer foundries, including Applied Nanotools Inc., which we employed for device fabrication in this work. In the multi-project wafer (MPW) process for large volumes of this foundry, the etch depth can be precisely controlled [46].

## 3 Fabrication and characterization

To experimentally verify the theoretical framework and simulated performance, MMOCs of C_1_ to C_4_ were fabricated on an SOI substrate comprising a 220-nm silicon waveguide layer and a 2-μm-thick buried oxide (BOX) through a 100 keV EBL system. The fabrication began with spin-depositing electron-sensitive resist followed by nanoscale patterning using the EBL technology to precisely define the MMOC device layouts. The patterned resist served as a mask for directionally controlled plasma etching with inductive coupling (ICP-RIE), which transferred the designed features into the silicon layer while maintaining subwavelength structural integrity. Finally, a 2.2-μm silicon dioxide cladding layer was deposited by using the plasma-enhanced chemical vapor deposition technology.

**Figure 6:**
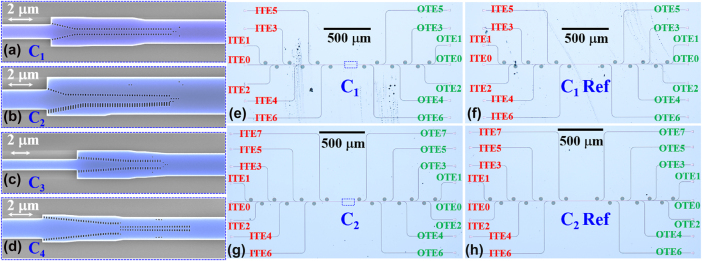
Pseudo-color SEM pictures of fabricated MMOCs (a) C_1_, (b) C_2_, (c) C_3_, and (d) C_4_. Microscope images of (e) device groups and (f) reference group for fabricated C_1_; microscope images of (g) device groups and (h) reference group for fabricated C_2_.

The fabricated C_1_, C_2_, C_3_, and C_4_ are given in Figure 6(a)–(d), respectively, where pseudo-color scanning electron microscopy (SEM) images are shown for better clarity. Figure 6(e)–(h) gives the microscope images of fabricated on-chip photonic integrated circuits with MMOCs of C_1_ and C_2_. Here, the on-chip measurement setup for an MMOC is composed of a converter group and reference group, in which the reference group has the same grating couplers, MUXs, DeMUXs, but without the corresponding converter. Both groups are fabricated on the same chip. Since two groups shown together are beyond the range of the used microscope, these groups are shown separately. The characterization methodology employs TEi/TMi MUX and DeMUX to establish the transmission measurement. For MUXs, the TE_0_/TM_0_ mode injected through input ports (labeled as ITEi/ITMi) is converted to TEi/TMi modes within the bus waveguide. Conversely, DeMUXs extract TEi/TMi modes from the bus waveguide and revert them to TE0/TM0 modes at output ports (denoted as OTEi/OTMi). Reference transmissions are determined by launching light into ITEi/ITMi ports, measuring optical power at OTEi/OTMi ports, then dividing these measurements by two to account for bidirectional conversion losses. Consequently, normalized transmission spectra for a specific mode conversion can be obtained by subtracting the spectra of the corresponding reference transmissions. Moreover, the on-chip measurement setups of converters C_3_ and C_4_ are shown in Fig. S4 (Supplementary Material).

**Figure 7:**
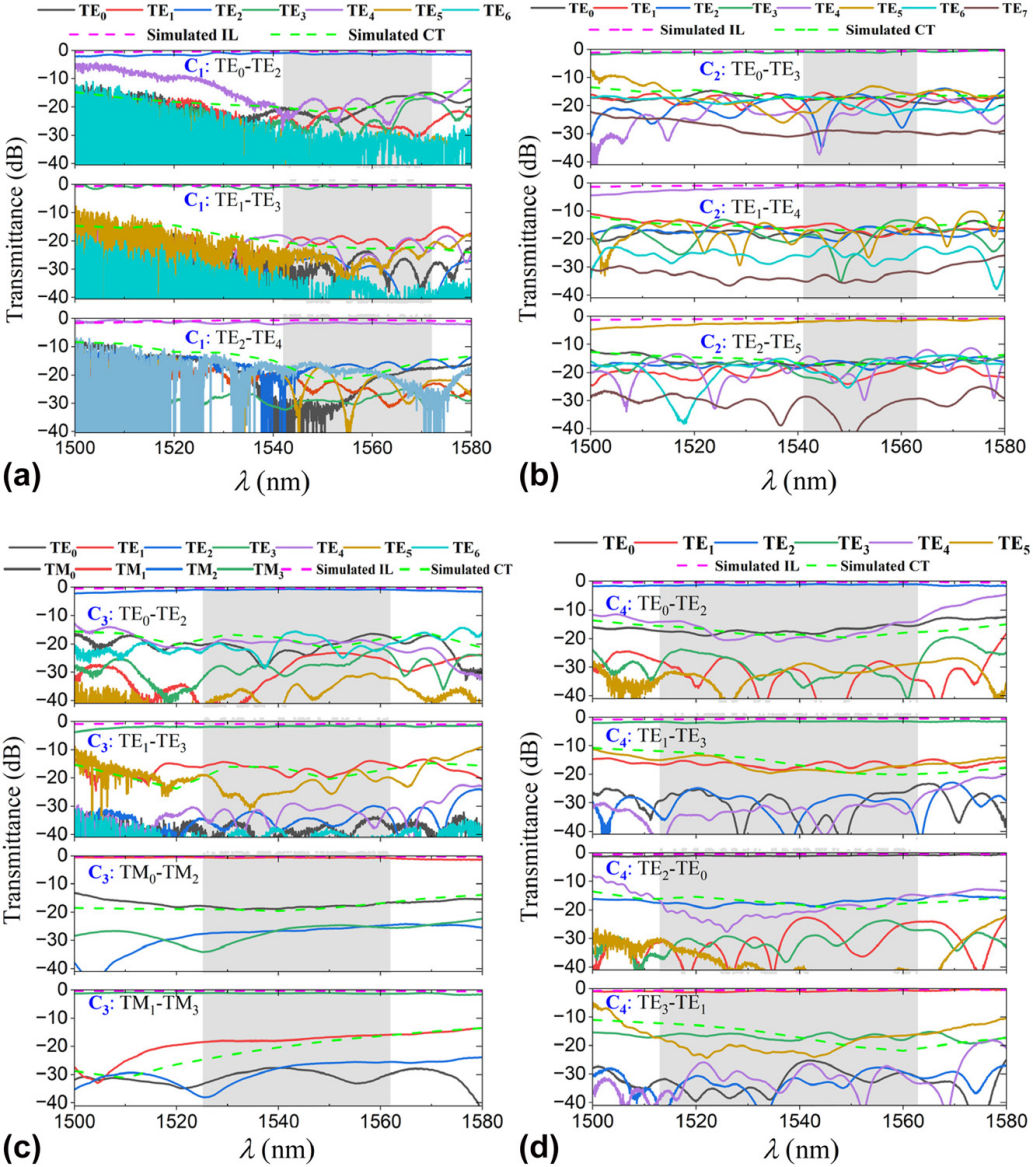
Measured transmittances spectra of fabricated MMOCs of (a) C_1_, (b) C_2_, (c) C_3_, and (d) C_4_, respectively, compared with simulated ILs and CTs.

The transmission spectra of fabricated MMOCs of C_1_–C_4_ were characterized by using a supercontinuum laser (NKT Photonics, SuperK EVO) and an optical spectrum analyzer (YOKOGAWA, AQ6375B). To highlight the comparison with many measured results, we use the magenta and green dashed lines to represent the simulation results. The transmittance of the desired mode is -IL_simulated_, and the highest transmittance of unwanted modes is −IL_simulated_ + CT_simulated_, respectively, in Figure 7. Deviations and deteriorations in measured ILs and CTs can be observed, especially in the wavelength range of 1,500–1,510 nm. Such differences in the simulated and measured results are mainly caused by unavoidable fabrication imperfections and the unexpected scattering from the waveguide sidewalls. Note that some CTs_simulated_ are somewhat higher than those in the measurements, such as the CT_simulated_ within the 1,505–1,550 nm wavelength range in Figure 7(d), which is caused by the blue-shifted central wavelength of the fabricated grating couplers. Moreover, one can see that the ILs and CTs of TE modes exhibit larger fluctuations over the 1,500–1,580 nm wavelength range. The reason is explained as follows. For TE modes, their eigenmode fields are mainly confined in the waveguide core. By contrast, the mode fields of the TM modes are concentrated in top/bottom surfaces of the waveguide. Thus, TE modes exhibit higher sensitivity to sidewall roughness in the SWGs structure of the MMOCs as well as in input/output grating couplers.

Measured spectral responses for converter C_1_ are displayed in Figure 7(a), demonstrating TE_0_-TE_2_, TE_1_-TE_3_, and TE_2_-TE_4_ conversions with respective ILs of 1.32 dB, 0.65 dB, and 1.53 dB, accompanied by CTs of −17.3 dB, −18.51 dB, and −15.5 dB at 1,550 nm. For maintaining the level of IL < 1.85 dB and CT < −12.5 dB across all three conversion channels, the operational bandwidth spans 30 nm (1,542–1,572 nm). From Figure 7(b), under the same IL and CT level, C_2_ operates across a 22-nm wavelength range (1,541–1,563 nm). As shown in Figure 7(c), converter C_3_ exhibits polarization-independent performance with measured IL/CT values of 0.77 dB/-18.67 dB (TE_0_-TE_2_), 1.42 dB/-19.62 dB (TE_1_-TE_3_), 0.6 dB/-17.85 dB (TM_0_-TM_2_), and 1.12 dB/-16.79 dB (TM_1_-TM_3_) at the central 1,550 nm wavelength. This quad-mode conversion maintains ILs < 1.85 dB and CTs < −12.5 dB across a 37-nm bandwidth (1,525–1,562 nm), which is larger than that of the dual-mode polarization-independent converter using the inverse design metastructure, under the same IL and CT requirements [44]. As to converter C_4_, measurements from Figure 7(d) show a 50-nm operational bandwidth (1,513–1,563 nm) for both TE_0_↔TE_2_ and TE_1_↔TE_3_ mode exchange, where both ILs and CTs are lower than those of metastructure enabled single-pair mode exchanger [29]. Such an MMOC achieves a two-fold improvement in mode exchange capacity over conventional mode exchangers. The overall measured performances of all fabricated MMOCs are summarized and shown in Table S2 (Supplementary Material). While we implemented this work on SOI substrates, the proposed multiple mode conversion concept remains applicable to alternative material systems including lithium niobate (LNOI) and silicon nitride (Si_3_N_4_) platforms across various optical bands.

Table 2 gives a systematic comparison with previous reports of metastructures based MMOCs. For those MMOCs with wide working bandwidths [23], [27], they can only operate for TE modes, showing polarization-dependent multiple mode conversions. The inverse designed metastructures exhibit high scalability, but the ILs are as large as 5.1 dB and 3.26 dB in [18], [47]. Moreover, optical power reflections can be observed in these MMOCs. The 2D metasurface design is also scalable, but an extra etching step for partially etched trenches is required [20]. By contrast, the proposed metastructures design framework exhibits large operating bandwidth for realizing ILs < 1.85 dB and CT < −12.5 dB, with compact device length ranged from 9.234 μm to 12 μm. Especially, compared with single mode exchanging device [27], the proposed C_4_ can double the mode exchange efficiency. Furthermore, the present MMOC C_3_ can break the polarization-dependent limit, showing great design flexibility of the proposed framework.

**Table 2:** Comparison with previous reported metastructure-based MMOCs.

Metastructure type	Function	PD/PI	Footprint (μm^2^)	Max IL (dB) @1,550 nm		Max CT (dB) @1,550 nm		Bandwidth (nm)^*c*^	
				Sim	Exp	Sim	Exp	Sim	Exp
Inverse design [18]	TE_0_/TE_1_-TE_2_/TE_3_	P	4 × 3	<1.2	<5.1	< −17.4	< −10.7	40 (IL < 1.2, CT < −17.4)	40 (IL < 5.1, CT < −10.7)
2D surface [20]	TE_0_/TE_1_-TE_2_/TE_3_	PD	20.9 × 2.4	0.48	–	−12.8	–	–	–
			TE_0_/TE_1_/TE_2_-TE_3_/TE_4_/TE_5_		16.2 × 3	<0.5	1@ 1,538	∼ −15	< −11.5	25 (IL < 1, CT < −15)	25 (IL < 3.5, CT < −11.5)
SWGs [23]	TE_0_/TE_1_-TE_1_/TE_0_	PD	1.3 × 2.7	∼0.19	∼0.22	∼-19	–	407 (IL < 0.65, CT < −10)	87 (CT < −12)
	TE_0_/TE_2_-TE_2_/TE_0_	PD	1.9 × 2.9	∼0.21	∼0.32	∼-32	–	340 (IL < 1.15, CT < −10)	87 (CT < −13.2)
	TE_1_/TE_2_-TE_2_/TE_1_	PD	3.7 × 2.1	0.45	–	−15.54	–	216 (IL < 0.85, CT < −10)	87 (IL < 1, CT < −8.1)
	TE_0_/TE_3_-TE_3_/TE_0_	PD	5.1 × 2.457	0.59	–	−12.6	–	159 (IL < 0.91, CT < −10)	80 (IL < 1, CT < −7.6)
SWGs [27]	TE_0_/TE_2_-TE_2_/TE_3_	PD	6.542 × 2.6	∼ < 0.3	–	∼ < −25	–	400 (IL < 0.9, CT < −10)	149 (IL < 0.4, CT < −18)
									150 (IL < 0.3, CT < −20)
Inverse design [47]	TE_0_/TE_1_/TE_2_-TE_1_/TE_2_/TE_0_	PD	3.84 × 12	< 2	–	< −21.9	–	100 (IL < 2.45, CT < −21.9)	50 (IL < 2.86, CT < −11.89)
		PD	3.84 × 9.6	–	–	–	–	80 (IL < 1.7, CT < −12.7)	50 (IL < 3.26, CT < −9.24)
This work	TE_0_/TE_1_/TE_2_-TE_2_/TE_3_/TE_4_	PD	10.499 × 2.38	0.55	1.53	−21.53	−15.5	60 (IL < 1, CT < −10.5)	30 (IL < 1.85, CT < −12.5)
	TE_0_/TE_1_/TE_2_-TE_3_/TE_4_/TE_5_	PD	11.1 × 3.134	0.77	1.5	−16.05	−16.34	80 (IL < 1, CT < −10.5)	22 (IL < 1.85, CT < −12.5)
	TE_0_/TE_1_/TM_0_/TM_1_-TE_2_/TE_3_/TM_2_/TM_3_	PI	9.234 × 2.898	0.86	1.42	−17.6	−16.79	100 (IL < 1, CT < −10.5)	37 (IL < 1.85, CT < −12.5)
	TE_0_/TE_1_/TE_2_/TE_3_-TE_2_/TE_3_/TE_0_/TE_1_	PD	12 × 2.23	0.5	1.39	−19.16	−16.44	90 (IL < 1, CT < −10.5)	50 (IL < 1.85, CT < −12.5)

–, Not mentioned. PD/PI, polarization-dependent/polarization-independent.

## 4 Dual-mode direct-access add/drop multiplexing system

**Figure 8:**
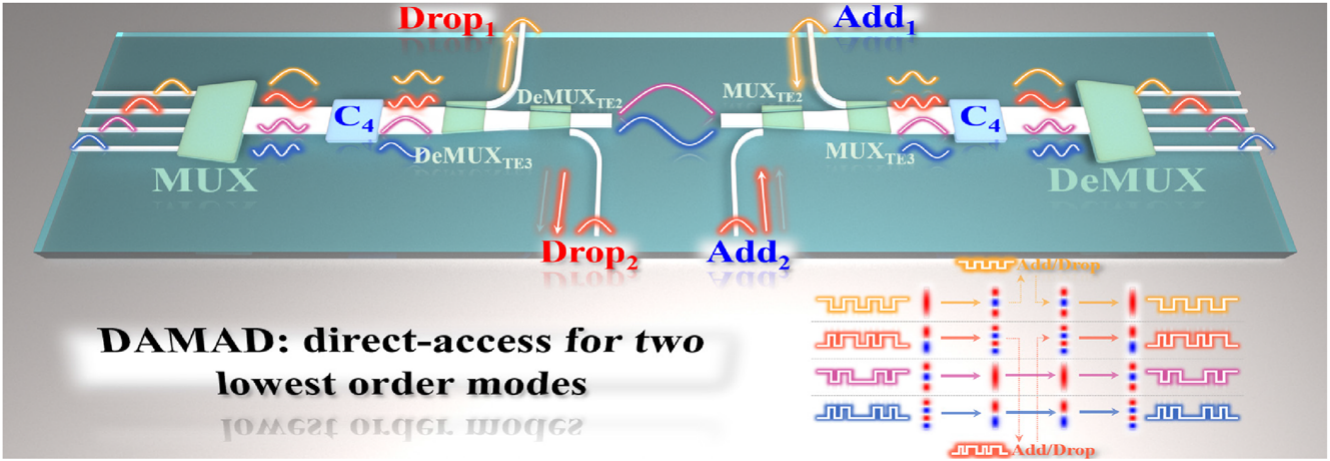
The TE_0_/TE_1_ dual-mode direct-access add/drop multiplexing system, where two MMOC C_4_ are used for doubling the mode exchanging efficiency.

The lower-order mode “direct-access” conundrum in multimode silicon photonics poses significant obstacles for selectively/efficiently manipulating desired lower-order modes (TE_0_, TE_1_) and building highly integrated systems such as mode-division multiplexing switches [48], [49]. Owing to the weak evanescent coupling strength and unwanted mode couplings induced by phase-matching conditions, the lower-order modes within a multimode bus waveguide are difficult to be accessed directly, especially the fundamental mode. To add/drop two lowest-order channels in a conventional MDM bus waveguide guiding TE_0_, TE_1_, TE_2_, and TE_3_ modes, two linked MUXs and DeMUXs for TE_2_ and TE_3_ modes are required to be used, which is an inefficient way to manipulate the lower-order modes and represents a substantial hindrance to the development of highly integrated on-chip multiplexing systems. The mode exchanger is an efficient solution for this conundrum [49]. However, two mode exchangers are required in this case, i.e., TE_0_↔TE_2_ and TE_1_↔TE_3_, which results in an increase in size and losses. By contrast, the designed converter C_4_, simultaneously realizing TE_0_↔TE_2_ and TE_1_↔TE_3_ dual-pair mode exchange, can double the mode exchanging efficiency. In this case, only one device is required to convert both TE_0_ and TE_1_ modes to higher-order modes for direct-access add/drop, greatly enhancing the system integration. The direct-access TE_0_/TE_1_ mode add/drop multiplexing system (DAMAD) is shown in Figure 8. MMOC C_4_ converts TE_0_ and TE_1_ modes to TE_2_ and TE_3_, respectively, and then these two modes are directly dropped to drop_1_ and drop_2_ ports by DeMUX_TE3_ and DeMUX_TE2_. Meanwhile, the TE_2_ and TE_3_ modes are converted to TE_0_ and TE_1_, respectively, which are guided in the bus waveguide. Reciprocally, modes launched from add_1_ and add_2_ ports are coupled into TE_2_ and TE_3_ modes in the bus waveguide via MUX_TE3_ and MUX_TE2_. After that, MMOC C_4_ restores those two modes to TE_0_ and TE_1_ modes, where TE_0_ and TE_1_ modes guided in the bus waveguide are restored to TE_2_ and TE_3_ modes. As such, the efficient direct access add/drop for the TE_0_/TE_1_ dual-mode is realized.

**Figure 9:**
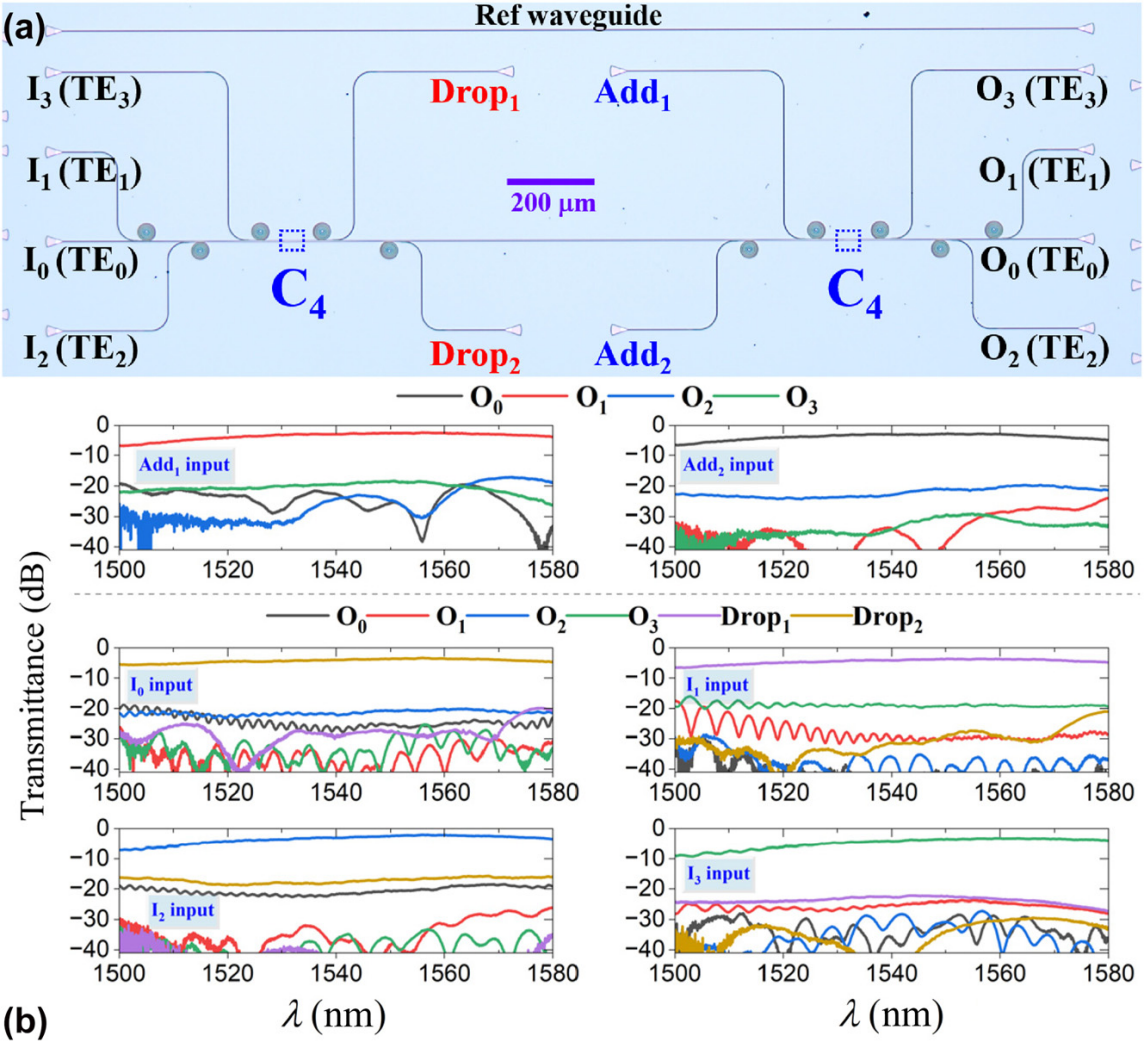
The experiments of the DAMAD: (a) The fabricated TE0/TE1 dual-mode direct-access add/drop multiplexing system. (b) Normalized measured transmissions at output ports of the fabricated direct-access add/drop multiplexing system, for different input ports.

**Figure 10:**
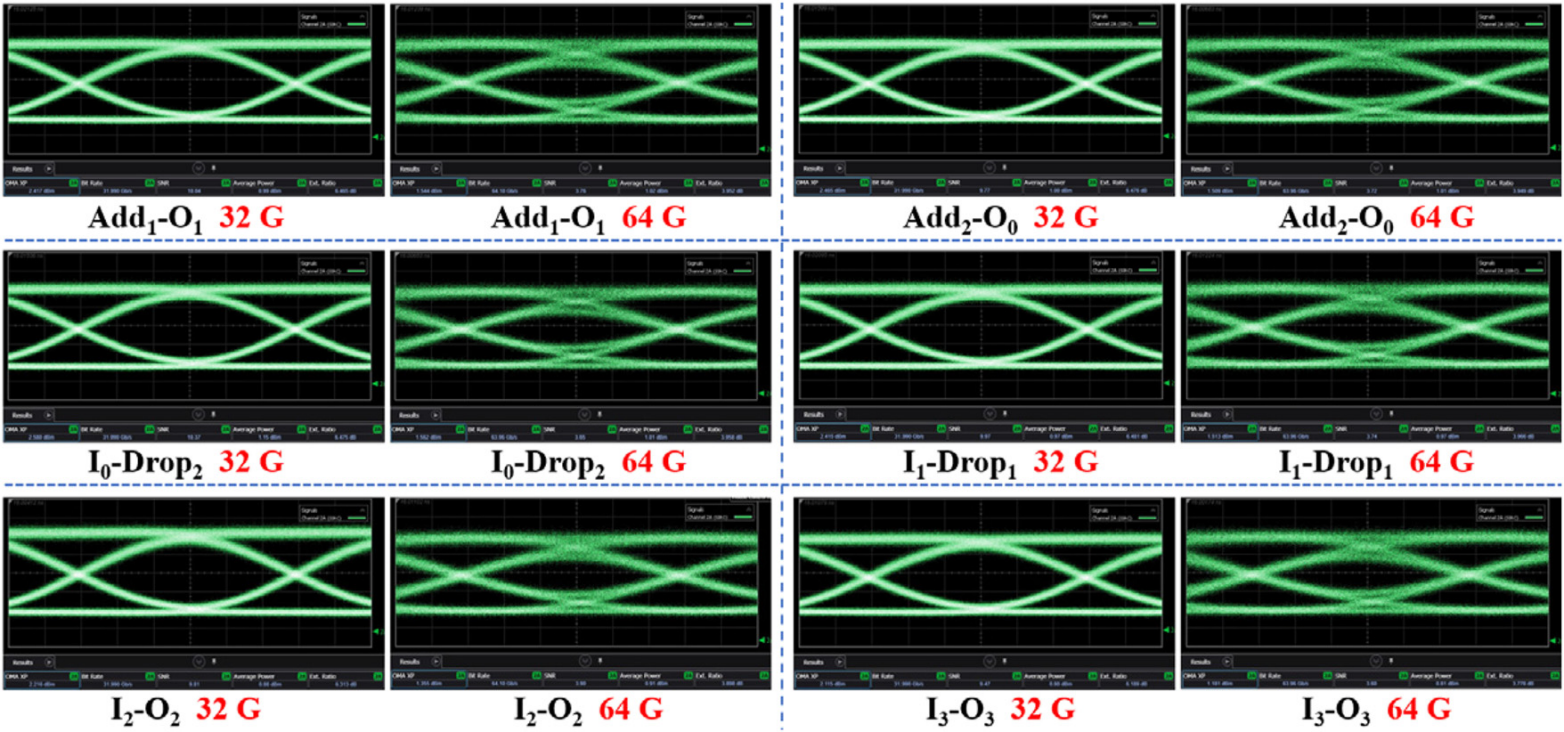
Measured eye diagrams for the fabricated DAMAD.

The TE_0_/TE_1_ dual-mode DAMAD was cofabricated with MMOCs of C_1_, C_2_, C_3_, and C_4_ on the same chip, as illustrated in Figure 9(a). The light from laser is coupled into I_1_, I_2_, I_3_, I_4_, Add_1_, and Add_2_ ports via input grating couplers, and the lights coupled out of O_1_, O_2_, O_3_, O_4_, Drop_1_, and Drop_2_ ports throughout grating couplers are recorded by an optical spectrum analyzer. A reference single-mode waveguide (namely Ref) was integrated adjacent to the system for transmission normalization. By subtracting the spectra of Ref waveguide, transmission spectra for each channel could be obtained. As shown in Figure 9(b), for input Add_1_ and Add_2_ ports, the add-mode operation for TE_0_ and TE_1_ modes achieves ILs below 3.5 dB and CT levels better than −17 dB across a 38-nm bandwidth (1,528–1,566 nm). As to the drop-mode operation for TE_0_ and TE_1_ modes and multiplexing-mode processes for TE_2_ and TE_3_ modes, for input I_1_–I_4_ ports, one can observe that the direct-access add/drop multiplexing system works well with ILs < 4.5 dB and CTs < −15.5 dB, over a 41-nm operational bandwidth spanning 1,535–1,576 nm. At the 1,550 nm central wavelength, measurements show ILs ranging from 2.49 to 3.68 dB with CTs between −22.5 and −17 dB. Furthermore, we measured the eye diagrams for the fabricated DAMAD, as shown in Figure 10. One can see that, the eye diagrams at central wavelength 1,550 nm of the fabricated DAMAD, under 32 and 64 Gbps of OOK modulations, demonstrate clear open eye diagrams for all add/drop and multiplexing channels. The measured extinction ratios exceed 6.1 dB and 3.7 dB for the 32 and 64 Gbps eye images, respectively. The experimental results validate that the proposed MMOC C_4_ achieves superior and reliable data communication capabilities.

To extend to higher-order multimode add/drop systems, a higher-order mode exchanger is required. For example, an MMOC that facilitates TE_2_↔TE_4_ and TE_3_↔TE_5_ conversions can broaden the operation scope to fifth-order mode. In such a configuration, the lower-order TE_2_ and TE_3_ modes can be direct-accessed. If the dual-pair mode exchange is not necessary in the DAMAD, the mode exchanger can be further designed to handle ultra-high-order modes, such as TE_10_ mode. For example, an MMOC performing TE_4_↔TE_10_ could be used to extract the TE_4_ mode for direct-access. Therefore, the direct-access for lower-order modes is not limited to TE_0_/TE_1_ modes, provided that a suitable mode exchanger is implemented.

## 5 Conclusions

In summary, we have proposed and experimentally verified a novel concept of scalable on-chip MMOCs enabled by the SWG metastructures. The MMOCs utilize SWG arrays to form synergistic coherent scattering and beam shaping regions within a taper-tailored multimode waveguide, thus enabling efficient target-supermode excitation and precise phase control of modal components within a single device. By optimizing the metastructure via the PSO method, multiple input modes can be converted into multiple output modes according to the functional requirements of the MMOC. The MMOCs exhibit superior performance in terms of low insertion losses, low intermodal crosstalks, and broad operational bandwidths. From simulation results, MMOCs of C_1_, C_2_, C_3_, and C_4_ show low ILs (0.3–0.86 dB) and low CTs (−16.05 to −21.87 dB) at central 1,550 nm wavelength. Specifically, the fabricated MMOCs of C_1_, C_2_, C_3_, and C_4_ achieve ILs below 1.85 dB and CTs below −12.5 dB across 50 nm in the best case. One exhibits polarization-independent mode conversion capability for TE_0_-TE_2_/TE_1_-TE_3_/TM_0_-TM_2_/TM_1_-TM_3_. To our knowledge, the first dual-pair mode exchanging MMOC is demonstrated, which facilitates simultaneous TE_0_↔TE_2_ and TE_1_↔TE_3_ mode exchanges. This dual-pair mode exchange feature doubles the mode exchange efficiency compared to conventional single-pair mode exchangers, thereby greatly enhancing the system integration and efficiency of lower-order mode DAMADs. Measured eye diagrams prove the high-speed data communication functionality of the proposed MMOC. These results highlight the potential of the proposed MMOCs to become key components in future high-integration multimode silicon photonic circuits, with significant implications for advancing optical communication and quantum computing technologies.

## Supplementary Material

Supplementary Materials include the optimized parameters of MMOCs C_1_, C_2_, C_3_, and C_4_, fabrication tolerance study, mode propagations of C_2_ in top view, mode propagations of C_5_ in top view, measurement setups of converters C_3_ and C_4_, and measurement results summarization.

## Supplementary Material

Supplementary Material Details
