# Supplementary material for: Metastructure-enabled scalable multiple mode-order converters: conceptual design and demonstration in direct-access add/drop multiplexing systems
Source: Nanophotonics. 2025 Dec 1;14(27):5117–32. doi: 10.1515/nanoph-2025-0364 (PMC12717911; doi:10.1515/nanoph-2025-0364)
Supplement: Supplementary file 1 — Supplementary Material Details [file j_nanoph-2025-0364_suppl_001.doc]

Supplementary Material

Metastructure-Enabled Scalable Multiple Mode-Order Converters: Conceptual Design and Demonstration in Direct-Access Add/Drop Multiplexing Systems

Zhenzhao Guo^1,3^, Weike Zhao^3^, Shengbao Wu^2^, Yunfeng Lai^1^, Shuying Cheng^1^, and Daoxin Dai^3^

*^1^Institute of Micro/Nano Devices and Solar Cells, School of Physics and Information Engineering, Fuzhou University, Fuzhou 350108, China*

*^2^Photonics Information Innovation Center, Hebei Provincial Center for Optical Sensing Innovations, College of Physics Science and Technology, Hebei University, Baoding 071002, China*

*^3^State Key Laboratory for Extreme Photonics and Instrumentation, College of Optical Science and Engineering, Zhejiang University, Hangzhou 310058, China*

**Corresponding author: gzhenzhao@fzu.edu.cn; sycheng@fzu.edu.cn*

1. Optimal parameters

MMOCs C_1_, C_2_, C_3_, and C_4_ are optimized by the PSO method, the optimal parameters (coordinates in this work) are summarized and shown in Table S1. The length is in units of μm, and period number *N*_1_, *N*_2_, *N*_3_, and *N*_4_ have no units.

**Table S1.** Coordinate and some key parameters of optimized MMOCs C_1_-C_4_, where coordinates in units of μm and *n* is period number with no units.

| C_1_ | *L*1 | *L*2 | *L*3 | *L*4 | *L*5 | *L*6 | *L*7 |
| --- | --- | --- | --- | --- | --- | --- | --- |
|  | (0, 0.6) | (2, 0.149) | (2.489, 0.149) | (23, 0.14) | (23, 0.14) | (0, 0.74) | (0, 1.19) |
|  | *L*8 | *L*9 | *L*10 | *L*11 | *L*12 | *L*13 | *L*14 |
|  | (2.21, 1.19) | (3.51, 1.147) | (6.65, 1.147) | (7.672, 0.976) | (9.8, 0.976) | (9.8, 0.28) | (23, 0.26) |
|  | *L*15 | *L*16 | *LN*2 | *LN*3 | *LN*4 | Λ_1_ | *a*_1_ |
|  | (2.489, 0.289) | (2, 0.289) | (10.399, 0.3) | − | (9.9, -0.05) | 0.2 | 0.1 |
|  | *N*1 | *N*2 | *N*3 | *N*4 | *w*t |  |  |
|  | 49 | 1 | 0 | 2 | 0.1 |  |  |
| C_2_ | *L*1 | *L*1’ | *L*2 | *L*2’ | *L*3 | *L*3’ | *L*4 |
|  | (0, 0.812) | (0, -0.812) | (1.5, 0.323) | (2.929, -0.278) | (1.588, 0.323) | (7.029, -0.278) | (10, 0.217) |
|  | *L*4’ | *L*5 | *L*5’ | *L*6 | *L*6’ | *L*7 | *L*7’ |
|  | (10, -0.172) | (10, 0.217) | (10, -0.172) | (0, 0.972) | (0, -1.162) | (0, 1.822) | (0, -1.312) |
|  | *L*8 | *L*8’ | *L*9 | *L*9’ | *L*10 | *L*10’ | *L*11 |
|  | (10, 1.822) | (4.745, -1.312) | (10, 1.822) | (5.238, -1.209) | (10, 1.822) | (5.438, -1.209) | (10, 1.822) |
|  | *L*11’ | *L*12 | *L*12’ | *L*13 | *L*13’ | *L*14 | *L*14’ |
|  | (8.038, -1.205) | (10, 1.822) | (10, -1.205) | (10, 0.377) | (10, -0.522) | (10, 0.377) | (10, -0.522) |
|  | *L*15 | *L*15’ | *L*16 | *L*16’ | *LN*2 | *LN*3 | *LN*4 |
|  | (1.588, 0.483) | (7.029, -0.628) | (1.5, 0.483) | (2.929, -0.628) | (10.699, 1.37) | (6.636, 1.12) | (11, 1.12) |
|  | *LN*3’ | *LN*2’ | *N*1 | *N*2 | *N*3 | *N*4 | *N*3’ |
|  | (10.059, 0.02) | (10, -0.205) | 50 | 1 | 2 | 1 | 4 |
|  | *N*2’ | Λ | *a* | *w*t |  |  |  |
|  | 2 | 0.2 | 0.1 | 0.103 |  |  |  |
| C_3_ | *L*1 | *L*2 | *L*3 | *L*4 | *L*5 | *L*6 | *L*7 |
|  | (0, 0.56) | (2, 0.439) | (2, 0.439) | (5.3, 0.229) | (8.5, 0.23) | (0, 0.827) | (0, 1.449) |
|  | *L*8 | *L*9 | *L*10 | *L*11 | *L*12 | *L*13 | *L*14 |
|  | (5.3, 1.449) | (6.1, 1.319) | (6.946, 1.319) | (9.136, 1.15) | (8.5, 1.15) | (8.5, 0.496) | (5.3,0.501) |
|  | *L*15 | *L*16 | *LN*2 | *LN*3 | *LN*4 | Λ | *a* |
|  | (2, 0.706) | (2, 0.706) | (7.635, 0.72) | (8.707, 0.158) | (8.934, -0.055) | 0.25 | 0.15 |
|  | *N*1 | *N*2 | *N*3 | *N*4 | *w*t for *N*2, *N*3 | *w*t for *N*4 |  |
|  | 34 | 1 | 1 | 2 | 0.1 | 0.11 |  |
| C_4_ | *L*1 | *L*2 | *L*3 | *L*4 | *L*5 | *L*6 | *L*7 |
|  | (0,0.82) | (5.3, 0.32) | (6.4, 0.32) | (6.249, 0.06) | (6.249, 0.06) | (0,1.001) | (0,1.115) |
|  | *L*8 | *L*9 | *L*10 | *L*11 | *L*12 | *L*13 | *L*14 |
|  | (1.8, 1.115) | (2.99, 1.015) | (5.947, 1.015) | (6.547, 0.915) | (12, 0.915) | (12, 0.242) | (6.249, 0.172) |
|  | *L*15 | *L*16 | *LN*2 | *LN*3 | *LN*4 | Λ | *a* |
|  | (6.4, 0.501) | (5.3, 0.501) | (9.2, 0.668) | (9.441, 0.649) | − | 0.2 | 0.1 |
|  | *N*1 | *N*2 | *N*3 | *N*4 | *w*t |  |  |
|  | 60 | 2 | 2 | 0 | 0.1 |  |  |
| C_5_ | *L*1 | *L*2 | *L*3 | *L*4 | *L*5 | *L*6 | *L*7 |
|  | (0, 1.565) | (6.8, 0.485) | (9.359, 0.485) | (12.5, 0.215) | (19.2, 0.215) | (0, 1.935) | (0, 2.365) |
|  | *L*8 | *L*9 | *L*10 | *L*11 | *L*12 | *L*13 | *L*14 |
|  | (0.3, 2.365) | (4.6, 2.364) | (5.9, 2.364) | (9.2, 2.244) | (19.2, 2.244) | (19.2, 0.585) | (12.5, 0.365) |
|  | *L*15 | *L*16 | *LN*2 | *LN*3 | *LN*4 | Λ_1_ | *a*_1_ |
|  | (9.359, 0.855) | (6.8, 0.855) | (21.444, 1.02) | (21.529, 0.598) | (19.9, -0.05) | 0.2 | 0.1 |
|  | *N*1 | *N*2 | *N*3 | *N*4 | *w*t |  |  |
|  | 96 | 10 | 4 | 4 | 0.1 |  |  |

1. Fabrication tolerance study

**
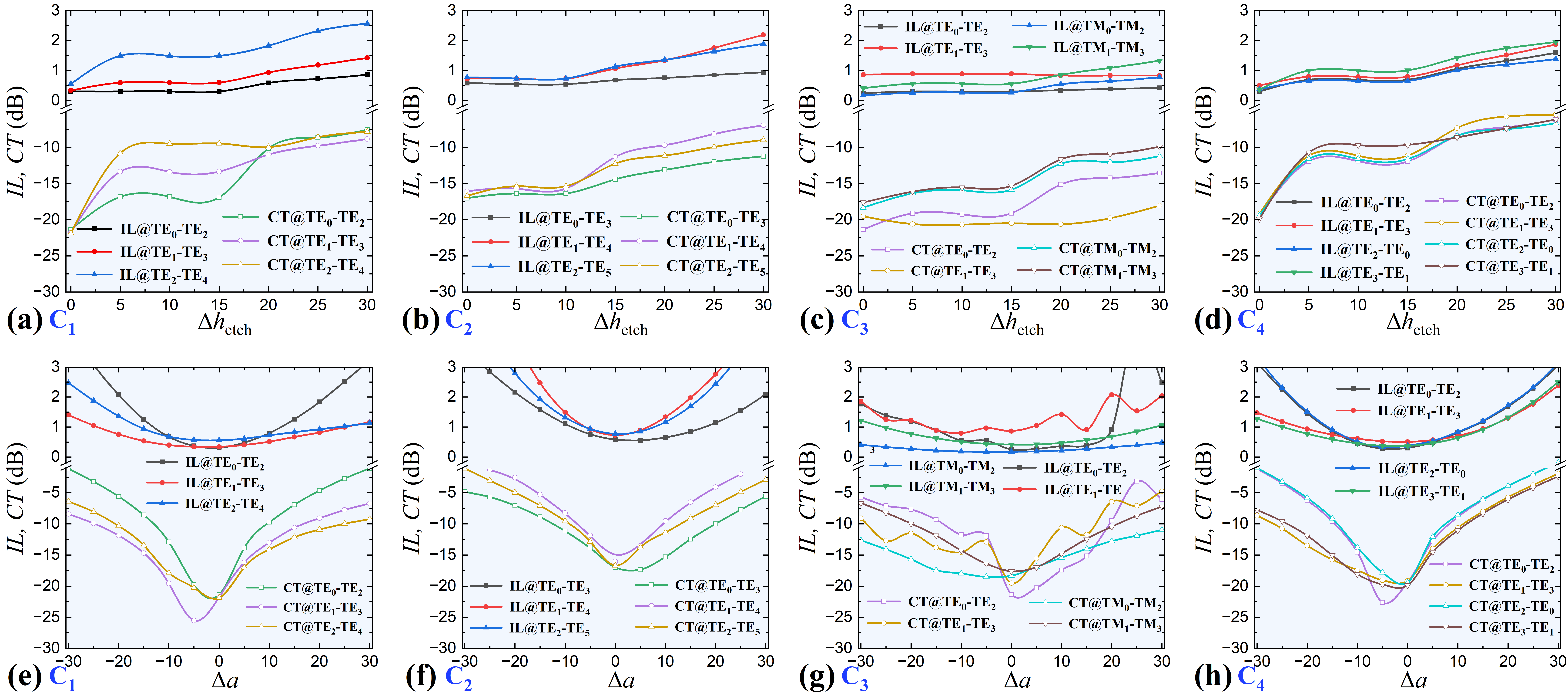
**

**Fig. S1.** Device tolerances of the under-etch variation ∆*h* for converters (a) C_1_, (b) C_2_, (c) C_3_, (d) C_4_, respectively; Device tolerances of the duty cycle variation ∆*a* for converters (e) C_1_, (f) C_2_, (g) C_3_, (h) C_4_, respectively.

1. Mode propagations of C_2_ in top view


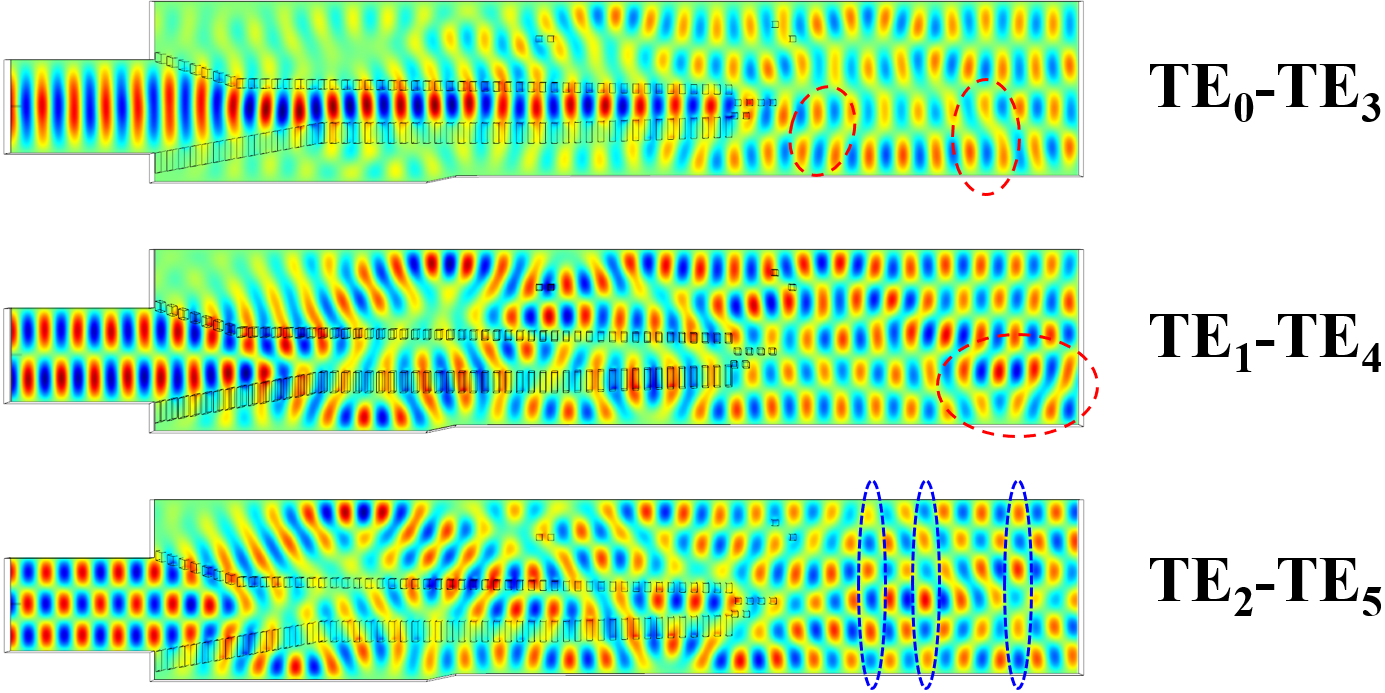


**Fig. S2** Simulated light propagations for MMOC C_2_, at the operating wavelength of 1.55 μm, in top view.

1. Mode propagations of C_5_ in top view


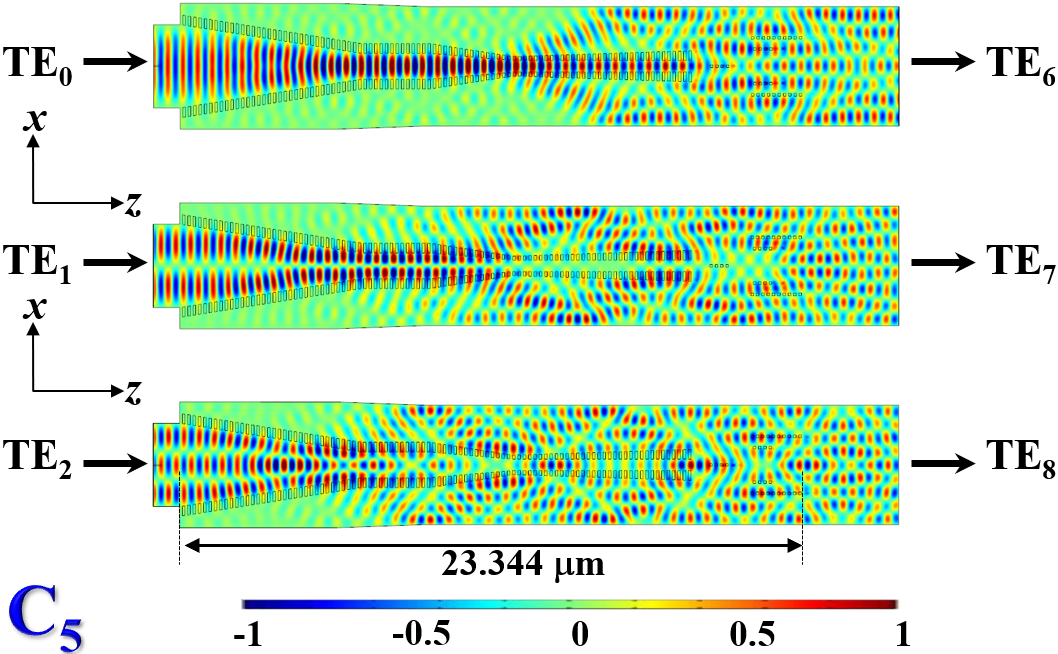


**Fig. S3** Simulated light propagations for TE_0_-TE_6_, TE_1_-TE_7_, and TE_2_-TE_8_ mode conversions, respectively, at the operating wavelength of 1.55 μm, in MMOC C_5_.

1. Measurement setups of converters C_3_ and C_4_

Figure S4 gives the microscope images of fabricated on-chip photonic integrated circuits with MMOCs of C_3_ and C_4_. For MMOC C_3_, a 7-mode-channel (De)MUX set with TE-type grating couplers is employed for characterizing TE_0_-TE_2_ and TE_1_-TE_3_ mode conversions. Meanwhile, a 4-mode-channel (De)MUX set with TM-type grating couplers is used for characterizing TM_0_-TM_2_ and TM_1_-TM_3_ mode conversions. The reason for less mode channels for TM modes compared with TE modes is that the number of eigen TM modes is less than that of the TE modes under the same waveguide width. Regarding MMOC C_4_, a 6-mode-channel (De)MUX set with TE-type grating couplers is applied here. As is observed, each measurement setup consists of a converter group and reference group. The reference group is identical to the converter group but without the corresponding converter. Moreover, we use spiral waveguide lines to effectively radiate out the residual power in the input access waveguide of the MUX, preventing unwanted reflections from the residual mode at the access waveguide facet which can cause large fluctuation in the measurements.


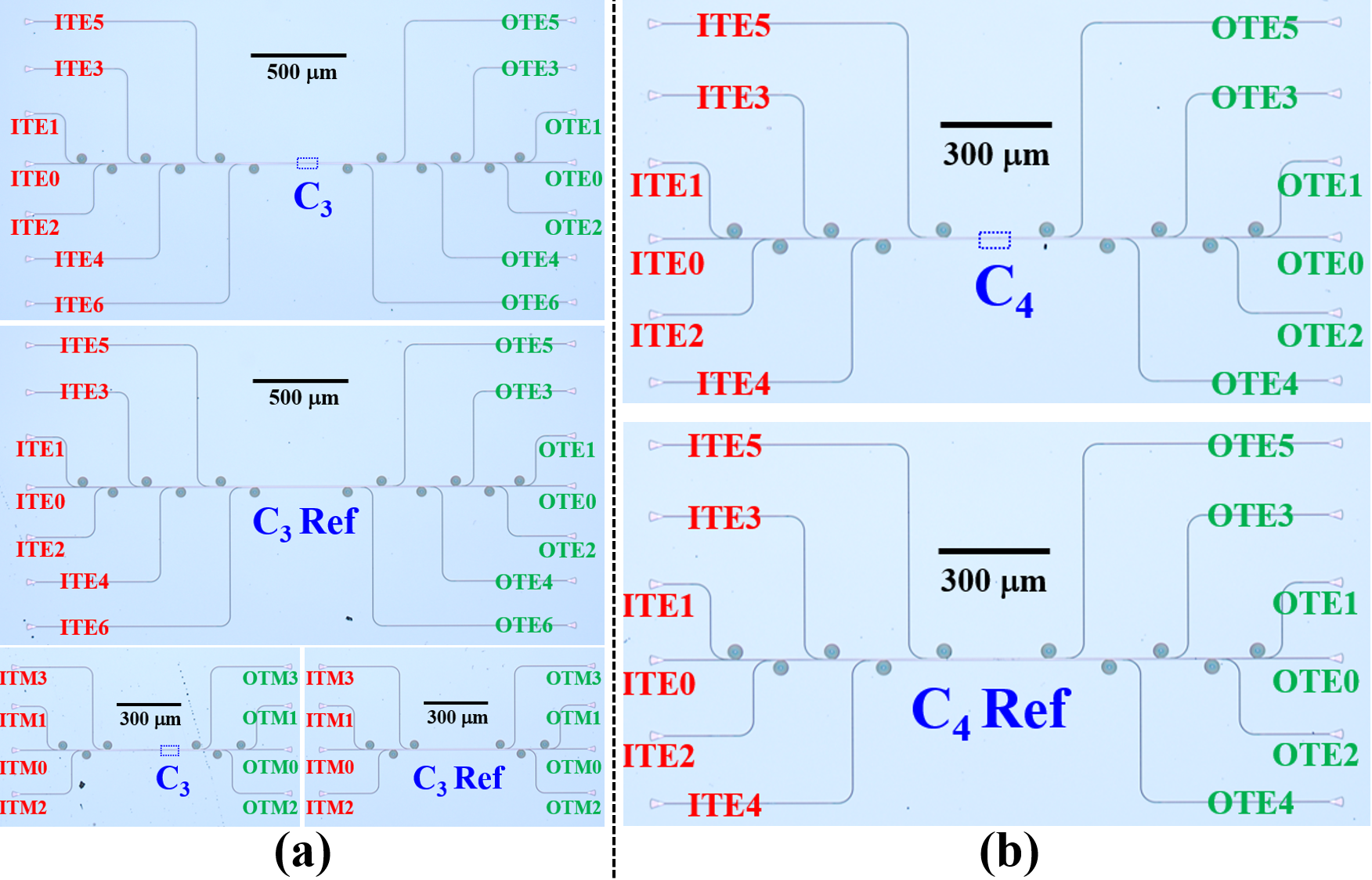


Fig. S4 Microscope images of measurement setup for MMOCs a) C3 and b) C4, respectively.

1. Measurement results summarization

For enhanced clarity, the measured ILs and crosstalk CTs of the fabricated MMOCs of C_1_, C_2_, C_3_, and C_4_ are comprehensively summarized and tabulated in Table S2, providing a consolidated reference for comparative performance analysis across operational bandwidths, for other works.

**Table S2.** Summarized measurement results of fabricated MMOCs of C_1_, C_2_, C_3_, and C_4_.

| MMOC | Function | IL@1550 nm (dB) | CT@1550 nm  (dB) | Working bandwidth  (IL < 1.85 dB and CT < -12.5 dB) | Device length  (μm) |
| --- | --- | --- | --- | --- | --- |
| C_1_ | TE_0_-TE_2_ | 1.32 | -17.3 | 1542-1572 nm | 10.499 |
|  | TE_1_-TE_3_ | 0.65 | -18.51 |  |  |
|  | TE_2_-TE_4_ | 1.53 | -15.5 |  |  |
| C_2_ | TE_0_-TE_3_ | 0.87 | -17.2 | 1541-1563 nm | 11.1 |
|  | TE_1_-TE_4_ | 1.17 | -17.51 |  |  |
|  | TE_2_-TE_5_ | 1.5 | -16.34 |  |  |
| C_3_ | TE_0_-TE_2_ | 0.77 | -18.67 | 1525-1562 nm | 9.234 |
|  | TE_1_-TE_3_ | 1.42 | -19.62 |  |  |
|  | TM_0_-TM_2_ | 0.6 | -17.85 |  |  |
|  | TM_1_-TM_3_ | 1.12 | -16.79 |  |  |
| C_4_ | TE_0_-TE_2_ | 1.24 | -16.44 | 1513–1563 nm | 12 |
|  | TE_1_-TE_3_ | 1.39 | -17.79 |  |  |
|  | TE_2_-TE_0_ | 0.89 | -16.81 |  |  |
|  | TE_3_-TE_1_ | 0.73 | -17.86 |  |  |
